# Treatment of Depression in Adolescents With Myasthenia Gravis

**DOI:** 10.7759/cureus.58408

**Published:** 2024-04-16

**Authors:** Mariana I Arce, Katherine A Breetz, Catherine A Martin

**Affiliations:** 1 Department of Child Psychiatry, University of Kentucky College of Medicine, Lexington, USA; 2 Department of Child Psychiatry, University of Kentucky, Lexington, USA

**Keywords:** suicide, physostigmine, myasthenia gravis, depression, adolescence

## Abstract

Myasthenia gravis (MG) is an autoimmune disorder in which, most commonly, there is a production of autoantibodies against the nicotinic acetylcholinergic receptors at the neuromuscular junction, resulting in skeletal muscle weakness. For pediatric patients, literature addressing the psychiatric implications of MG and suitable treatment options for individuals with concurrent psychiatric illnesses is scarce. In this case report, an adolescent with MG and comorbid depression was treated following a suicide attempt via self-poisoning. The patient experienced an improvement of depressive symptoms upon initiating fluoxetine, despite concerns raised by previous studies suggesting that fluoxetine might block acetylcholine receptors at the neuromuscular junction with varying degrees of affinity, potentially worsening MG symptoms. In this case, our patient exhibited sustained control of her MG symptoms without exacerbation once she was started on fluoxetine. This case highlights the value of further investigation into the safety and efficacy of selective serotonin reuptake inhibitors (SSRIs) in the management of depression among pediatric patients with MG.

## Introduction

Myasthenia gravis (MG) is a neuromuscular disorder with an incidence ranging from 1.7 to 30.0 cases per million person-years in adults. In pediatric patients, incidence rates are markedly lower at approximately 1.0 to 5.0 cases per million person-years [1]. The most common form is due to the production of autoantibodies against the nicotinic acetylcholinergic receptors at the neuromuscular junction, thus causing skeletal muscle weakness. MG is associated with several comorbidities, including severe depressive symptoms and anxiety, which are "high even when compared to other autoimmune conditions" [2]. In adults, the prevalence of depression is 36%, and there is an association with an increased risk of suicide attempts by self-poisoning [2,3]. This is in part attributed to the social and physiologic challenges that are experienced by this patient population, namely the symptoms of fatigue associated with MG [1,4]. As the coexistence of MG and depression with suicidal ideation is rare in adolescents, limited information is available regarding effective treatment recommendations [5]. Furthermore, literature describing potential interactions between selective serotonin reuptake inhibitors (SSRIs) and medications utilized to manage MG is scarce, further challenging clinical decisions. The following case report describes a treatment approach that proved successful in the management of depression in a pediatric patient with MG.

## Case presentation

A 13-year-old female presented to the emergency department following suicidal ideation and self-poisoning. Her father reported access to diphenhydramine medication, and 72 pills were missing. The morning after the incident, her father brought her to the hospital, initially mistaking her sedation and lack of movement for an MG crisis. She was subsequently transferred to the children’s hospital for stabilization and then transferred to the Behavioral Health Unit (BHU). Two days after admission, she completed the Children’s Depression Inventory and displayed a very elevated T-score of 75. She and her father also completed the Child Behavior Checklist. Elevations on the Youth Self-Report Diagnostic and Statistical Manual of Mental Disorders (DSM)-oriented scales included affective, attention-deficit hyperactivity disorder, and post-traumatic stress disorder (PTSD). On the caregiver Child Behavior Checklist, the following DSM-oriented scales were elevated: anxiety and PTSD Problems. While initially on the unit, she exhibited a flat affect and reported intentionally ingesting excessive medication in an attempt to prolong sleep, wishing she would die. She displayed indifference and minimal participation during the initial days of admission. She later revealed a chronic history of self-harm, specifically cutting her arms, and long-term thoughts of suicide but no previous plan. Before this hospital presentation, she had been engaged in outpatient therapy and prescribed an SSRI. However, the father later found the full bottle of the SSRI since she had not taken it.

Given the patient’s presentation of suicidal ideation with subsequent self-poisoning, the patient was started on fluoxetine and admitted to the BHU. Within a few days of initiation of fluoxetine, the patient’s mood, sleep, and appetite began to improve. She also became more motivated to participate in her care and more engaged in individual, group, and family therapy sessions. She also reported cessation of suicidal ideation. Being several half-lives (2-10 hours) out from the diphenhydramine overdose may have also contributed to early improvement in some of her symptoms. Throughout her admission, the patient’s MG remained well-controlled without an exacerbation of symptoms after the initiation of fluoxetine. The patient was able to continue with fluoxetine 10 mg and pyridostigmine 180 mg at discharge. The patient was also scheduled to participate in continued therapy following discharge.

**Figure 1:**
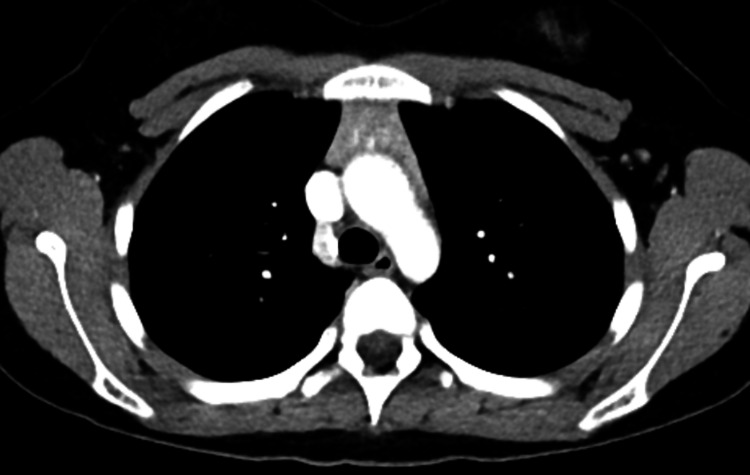
Axial CT chest with IV contrast

On review of her record, the following history regarding the onset, assessment, and treatment of her MG was obtained. The patient's MG symptoms first manifested at age 11 with slurred speech, fatigue, and muscle weakness that worsened throughout the day. Her school nurse ultimately saw her as experiencing increased difficulty with tasks that “used to be easy” for her, such as sustained speech, and struggling with keeping her eyes open throughout the school day. The patient was later admitted to the hospital where she was diagnosed with MG after testing confirmed that she was seropositive for both acetylcholine receptor and MuSK antibody titers. She also displayed persistent signs of dysphagia. The patient was started on oral pyridostigmine 180 mg, which provided substantial relief of her symptoms. She underwent a chest CT, which revealed an enlarged, heterogenous thymus, which was later identified as a thymoma and was surgically removed (Figure 1). Following thymectomy, the patient was able to begin tapering off her pyridostigmine and return to relatively normal baseline functioning. However, soon afterward, she was diagnosed with COVID-19, and she experienced an exacerbation of her MG requiring a higher dose of pyridostigmine.

Written consent and assent for publication of this case report were obtained from the patient and the parent of the patient.

## Discussion

Our patient’s presentation of depression complicated by suicidal ideation and subsequent self-poisoning emphasizes the importance of further research regarding the treatment of depression in this vulnerable patient population. To date, there is limited clinical guidance on treating adolescents with this comorbidity.

Given that fluoxetine is recognized as an FDA-approved, first-line agent for treating depression in pediatric and adolescent patients, we decided to start our patient on fluoxetine for the management of her depressive symptoms. However, this decision was made with consideration of studies suggesting a potential for pharmacological interactions between fluoxetine and pyridostigmine. Fluoxetine is capable of blocking acetylcholine receptors at the neuromuscular junction (NMJ) with varying degrees of affinity [6,7]. Pyridostigmine is also known to act at this site by inhibiting the acetylcholinesterase (AChE) enzyme, thus increasing the amount of acetylcholine available for electrical signaling between nerves and muscles, resulting in an improvement of muscle fatigue symptoms associated with MG. These findings suggest the possibility for pyridostigmine and fluoxetine to have opposing actions at the neuromuscular junction. There is a need for further research on the possible interactions between SSRIs and medications used to manage MG as both have benefits in the effective management of depressive symptoms associated with this autoimmune condition.

This case underscores how pediatric patients who suffer from MG and comorbid depression can benefit from treatment targeting both conditions. Additionally, our patient seemed to benefit from engaging in individual, group, and family therapy sessions during her hospitalization, suggesting that further investigation is warranted in the multimodal approaches that can be utilized in the treatment of depression in these patients. This case emphasizes the need for comprehensive assessment and management of mental health symptoms in pediatric patients with MG. Further research is warranted to explore optimal treatment strategies for depression in pediatric patients with MG, considering potential interactions with medications used to manage the autoimmune condition.

## Conclusions

This case highlights the successful management of depression with self-poisoning in a pediatric patient with MG. Fluoxetine, an SSRI, proved effective in improving the patient's depressive symptoms without adversely affecting her MG. The importance of combining an SSRI with therapy and continued care by neurology is also underscored in this depressed adolescent. Additionally, this case underscores the importance of routinely assessing depression and anxiety in pediatric patients with MG.
